# Distribution of erlotinib in rash and normal skin in cancer patients receiving erlotinib visualized by matrix assisted laser desorption/ionization mass spectrometry imaging

**DOI:** 10.18632/oncotarget.24928

**Published:** 2018-04-06

**Authors:** Meiko Nishimura, Mitsuhiro Hayashi, Yu Mizutani, Kei Takenaka, Yoshinori Imamura, Naoko Chayahara, Masanori Toyoda, Naomi Kiyota, Toru Mukohara, Hiroaki Aikawa, Yasuhiro Fujiwara, Akinobu Hamada, Hironobu Minami

**Affiliations:** ^1^ Division of Medical Oncology/Hematology, Kobe University Graduate School of Medicine, Kobe, Hyogo, Japan; ^2^ Division of Molecular Pharmacology, National Cancer Center Research Institute, Tokyo, Japan; ^3^ Division of Clinical Pharmacology and Translational Research, Exploratory Oncology Research and Clinical Trial Center, National Cancer Center, Tokyo, Japan; ^4^ Present Address: Department of Medical Oncology, Kobe Minimally invasive Cancer Center, Kobe, Hyogo, Japan; ^5^ Cancer Center, Kobe University Hospital, Kobe, Hyogo, Japan; ^6^ Department of Breast and Medical Oncology, National Cancer Center Hospital, National Cancer Center, Tokyo, Japan; ^7^ Department of Medical Oncology and Translational Research, Graduate school of Medical Sciences, Kumamoto University, Kumamoto, Japan

**Keywords:** erlotinib, rash, mass spectrometry imaging, drug distribution, liquid chromatography-tandem mass spectrometry

## Abstract

**Background:**

The development of skin rashes is the most common adverse event observed in cancer patients treated with epidermal growth factor receptor-tyrosine kinase inhibitors such as erlotinib. However, the pharmacological evidence has not been fully revealed.

**Results:**

Erlotinib distribution in the rashes was more heterogeneous than that in the normal skin, and the rashes contained statistically higher concentrations of erlotinib than adjacent normal skin in the superficial skin layer (229 ± 192 vs. 120 ± 103 ions/mm^2^; *P* = 0.009 in paired *t*-test). LC-MS/MS confirmed that the concentration of erlotinib in the skin rashes was higher than that in normal skin in the superficial skin layer (1946 ± 1258 vs. 1174 ± 662 ng/cm^3^; *P* = 0.028 in paired *t*-test). The results of MALDI-MSI and LC-MS/MS were well correlated (coefficient of correlation 0.879, *P* < 0.0001).

**Conclusions:**

Focal distribution of erlotinib in the skin tissue was visualized using non-labeled MALDI-MSI. Erlotinib concentration in the superficial layer of the skin rashes was higher than that in the adjacent normal skin.

**Methods:**

We examined patients with advanced pancreatic cancer who developed skin rashes after treatment with erlotinib and gemcitabine. We biopsied both the rash and adjacent normal skin tissues, and visualized and compared the distribution of erlotinib within the skin using matrix-assisted laser desorption/ionization mass spectrometry imaging (MALDI-MSI). The tissue concentration of erlotinib was also measured by liquid chromatography-tandem mass spectrometry (LC–MS/MS) with laser microdissection.

## INTRODUCTION

The development of molecular-targeted compounds has advanced into the clinical stages to accelerate precision medicine. However, it is also recognized that distinct adverse effects associated with the inhibition of target molecules frequently develop. With regard to drug development, the majority of clinical failures have been due to a lack of either efficacy or safety. And according to the classification of therapeutic areas, oncology had the highest clinical failure rate [1–5]. The proportion of oncology drug failure due to safety is increasing [4], so it is important to elucidate the pharmacological mechanisms underlying on-target and off-target adverse effects. In addition, the development of mechanism-based management of adverse effects will be beneficial to patients.

Erlotinib is an effective human epidermal growth factor receptor-tyrosine kinase inhibitor (EGFR-TKI), which was approved for the treatment of advanced pancreatic cancer in combination with gemcitabine as well as for other malignant tumors such as lung cancer [6, 7]. Rash, follicular papulopustular eruption, or acne-form eczema on the face, scalp, chest, and upper back are the most common and cumbersome adverse events, and were observed in 72–93% of advanced pancreatic cancer patients treated with gemcitabine plus erlotinib, and in 73–98% of patients with non-small cell lung cancer treated with erlotinib monotherapy [6, 8–10]. Interestingly, a significant relationship between rash development and survival has been reported in clinical trials [6, 8, 11]. However, the rash may become severe in some cases, resulting in the discontinuation or dose reduction of erlotinib [12]. The pathogenesis of erlotinib-induced rash has not been fully elucidated [13].

Liquid chromatography-tandem mass spectrometry (LC-MS/MS) can accurately determine the average concentration of a compound in plasma or homogenized tissues; however, it cannot provide information on the spatial distribution of a compound [14, 15]. Mass spectrometry imaging (MSI) has the ability to provide spatial information on the distribution of molecules in a tissue section, including exogenous substances, while maintaining the ability of MS to identify the molecules [14, 16]. Among several methods for molecular ionization that have different efficiencies for the range of molecular types of interest, matrix-assisted laser desorption/ionization (MALDI)-MSI analysis is the most advanced in development, and is enable to detect a wide range of analytes with high spatial resolution. Because it has the potential to analyze drug distribution in a tissue [17], MALDI-MSI has been applied as an innovative tool in pre-clinical cancer studies [18–21].

We conducted a prospective clinical study to visualize the distribution of erlotinib within the skin rash of patients with advanced pancreatic cancer. The differences in erlotinib concentrations in the tissues of normal skin and rash were investigated using MALDI-MSI and LC-MS/MS in combination with laser microdissection (LMD).

## RESULTS

### Assessment of clinical characteristics

Between December 2013 and August 2015, five patients were enrolled in this study. All of the patients were male, and the median age was 55 years old (range, 51 to 70 years) (Supplementary Table 1). The median duration from the start of treatment to biopsy was 2.0 months (range, 0.75 to 4.0 months), and the median elapsed time from final oral administration of erlotinib to biopsy was 3.0 h (range, 2.0 to 6.0 h). The grade of skin rash was 2 in three patients and 1 in two patients (Supplementary Figure 1). No dose reduction or skipping of erlotinib or gemcitabine treatment occurred in any patient. Of the enrolled patients, three had stable disease and two patients had progressive disease.

### Erlotinib concentrations in plasma and skin tissues

The erlotinib concentration in plasma was 1596 ± 953 ng/mL (mean ± standard deviation [SD], Supplementary Table 1). The erlotinib concentration in the entire tissue section, as measured by LC-MS/MS, was higher in the skin rash than in normal skin (3180 ± 1529 vs. 2549 ± 1456 ng/cm^3^), although the difference was not statistically significant (*P* = 0.0637, Supplementary Figure 2). Compared to normal skin, more inflammatory cells infiltrated the skin rash (*P* = 0.0042, Supplementary Figure 3), and a thickened epidermis, irregular elongation of the rete ridge, and intercellular edema were observed. Immunohistochemistry data did not show an obvious difference in EGFR expression between normal skin and skin rash (Supplementary Figure 3). MALDI-MSI of the entire tissue section showed that the rash tended to have a higher concentration of erlotinib than the normal skin (145 ± 62 ions/mm^2^ vs. 112 ± 69 ions/mm^2^, *P* = 0.052, Supplementary Figure 2).

### Comparison of erlotinib focal distribution in normal skin and rash using MALDI-MSI

**Figure 1:**
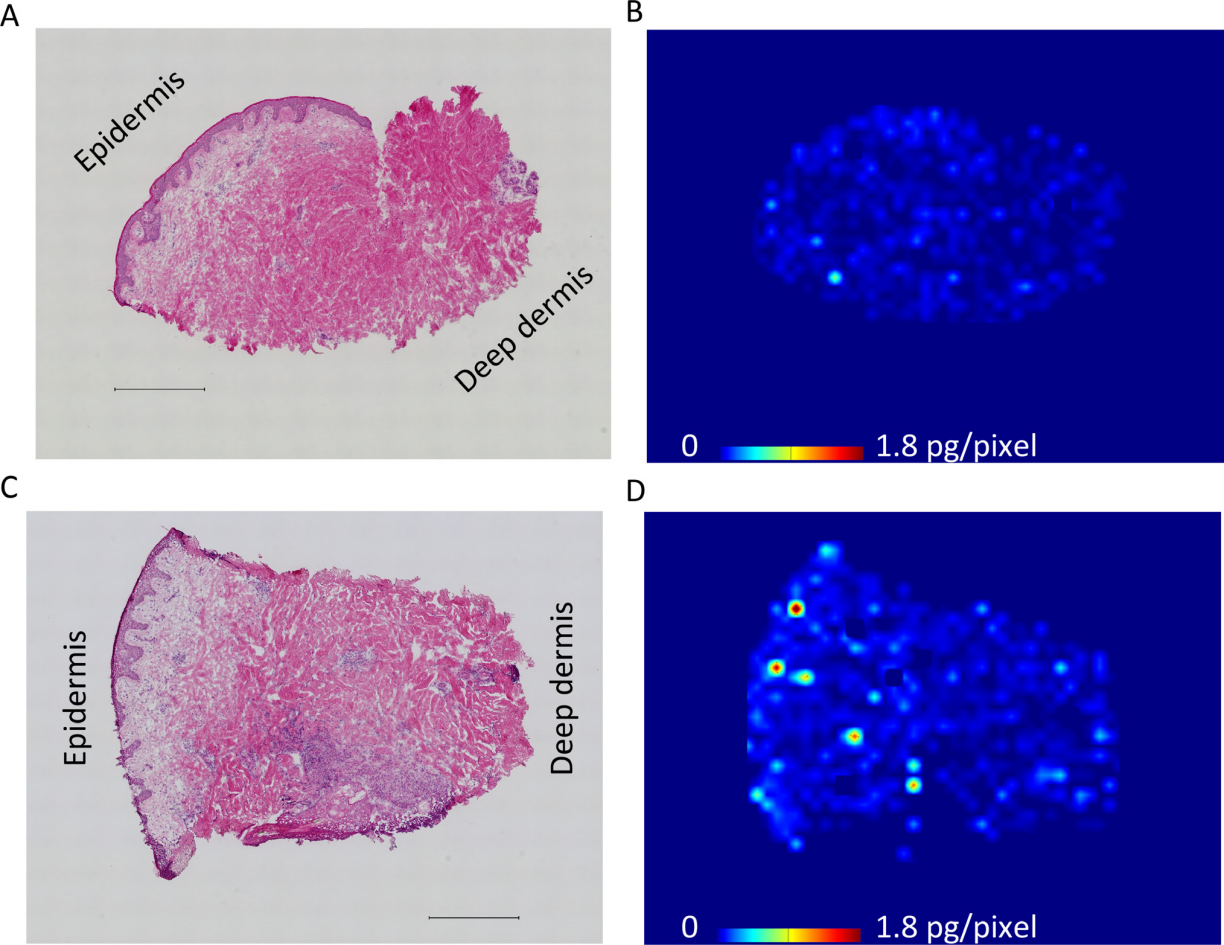
Representative molecular images of erlotinib distribution in skin rash and adjacent normal skin (**A**) Hematoxylin and eosin staining of the adjacent normal skin including epidermis to deep dermis layers, which were concurrently collected at the time of rash biopsy. Scale bar = 500 μm. (**B**) Determination of erlotinib distribution in the normal skin by mass spectrometry imaging. Molecular images were acquired at a step size of 60 μm. Scale bar indicates erlotinib quantity, pg/pixel. (**C**) Hematoxylin and eosin staining of the rash, showing that inflammatory cells infiltrated into the papillary dermis and superficial-reticular dermis. Scale bar = 500 μm. (**D**) Molecular image of erlotinib distribution in the rash, indicating that erlotinib was predominantly localized in the superficial layer of the skin.

**Figure 2:**
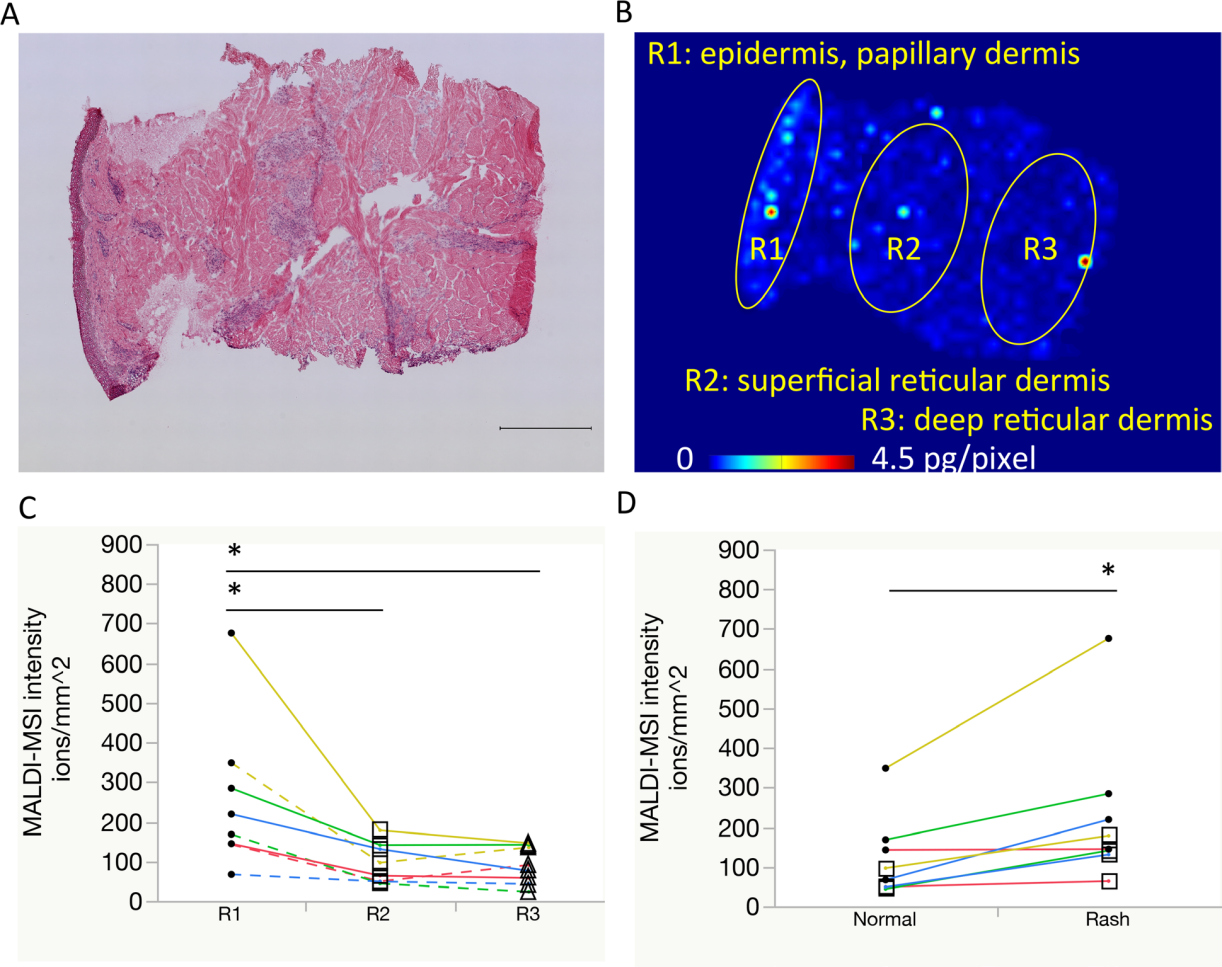
Comparison of erlotinib focal distribution using mass spectrometry imaging (**A**, **B**) Representative images of the skin rash: hematoxylin and eosin staining with a scale bar of 500 μm, and molecular image of erlotinib distribution, respectively. Regions of interest are as follows: R1, epidermis to papillary dermis layer; R2, superficial reticular dermis layer; R3, deep reticular dermis layer. (**C**) Erlotinib focal concentrations were compared among R1 (circle), R2 (square), and R3 (triangle). Same color line indicates same patient. Solid line indicates rash and dotted line indicates normal skin. (**D**) Erlotinib focal concentrations within the superficial skin layer (R1 with R2) were compared between the normal skin and rash using molecular images of erlotinib. One patient's paired samples that was insufficient in quantity for focal distribution analysis were excluded. ^*^P < 0.05.

We investigated erlotinib localization in the skin layer using MALDI-MSI. The distribution of erlotinib was more heterogeneous in the skin rash compared to the normal skin (Figure 1, Supplementary Figure 4, and Supplementary Figure 5). Within the skin structure (Figure 2), erlotinib was highest in the epidermis–papillary dermis (R1) compared with the superficial- (R2) and deep-reticular dermis layers (R3) (256 ± 191, 94 ± 51, and 89 ± 47 ions/mm^2^ in R1, R2, and R3, respectively; *P* = 0.030 for R1 vs. R2, and *P* = 0.025 for R1 vs. R3 in Tukey-Kramer HSD test). When the focal distribution of erlotinib was compared between the skin rash and normal skin, it was found that the rash had significantly higher erlotinib concentrations than the normal skin (229 ± 192 vs. 120 ± 103 ions/mm^2^; *P* = 0.009, Figure 2) in the superficial skin layer (R1 and R2 in total). The tissue plasma ratio, which was the relative value of erlotinib ion intensity divided by the matched plasma concentration, was also significantly higher in the rash compared to the normal skin (0.13 ± 0.07 vs. 0.07 ± 0.05, *P* = 0.006) (Supplementary Figure 6). There were no significant differences in erlotinib concentrations between the normal skin and rash in the deep skin layer (R3) (Supplementary Figure 6).

### Difference in erlotinib concentration between normal skin and rash by LMD

**Figure 3:**
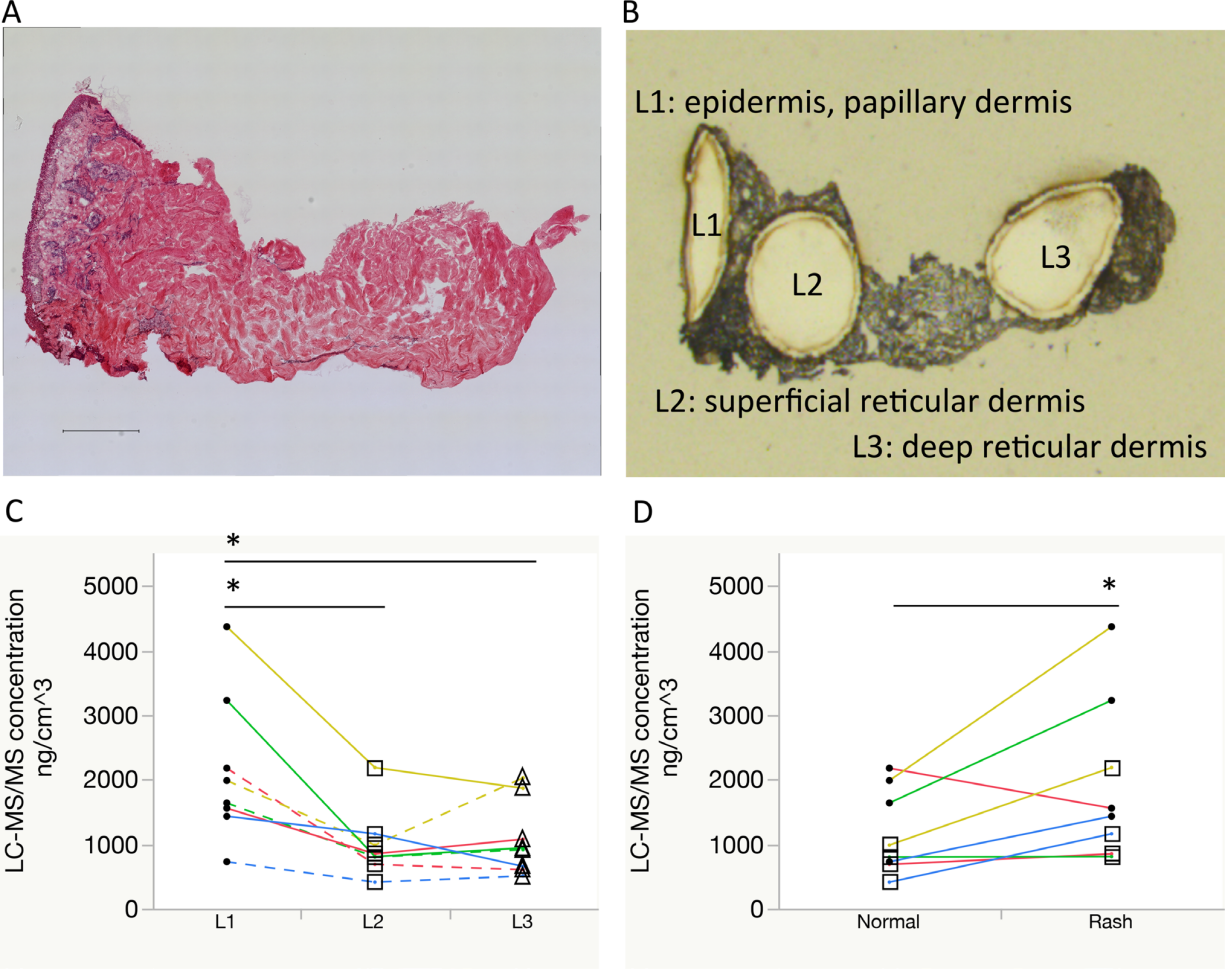
Comparisons of erlotinib focal concentrations using laser microdissection and liquid chromatography-tandem mass spectrometry (**A**, **B**) Representative images of the rash: hematoxylin and eosin staining with a scale bar of 500 μm and optical image after laser microdissection, respectively. Localization of the dissected pieces is as follows: L1, epidermis to papillary dermis layer; L2, superficial reticular dermis layer; L3, deep reticular dermis layer. (**C**) Erlotinib focal concentrations were compared between L1 (circle), L2 (square), and L3 (triangle). Same color line indicates same patient. Solid line indicates rash and dotted line indicates normal skin. (**D**) Erlotinib focal concentrations within the superficial layer (L1 with L2) were compared between the normal skin and rash. One patient's paired samples that was insufficient in quantity for focal distribution analysis were excluded. ^*^P < 0.05.

To confirm the results of focal MALDI-MSI according to skin layer, we performed regional LC-MS/MS of erlotinib concentrations using the LMD method (Figure 3). In comparing the epidermis–papillary dermis (L1), superficial- (L2), and deep reticular dermis (L3) regions, erlotinib concentration was found to be significantly higher in the L1 than L2 or L3 regions (2136 ± 1149, 984 ± 531, and 1072 ± 572 ng/cm^3^ in L1, L2, and L3, respectively, in both normal skin and skin rash; *P* = 0.024 for L1 vs. L2, and *P* = 0.038 for L1 vs. L3 in Tukey-Kramer HSD test). When the focal concentration of erlotinib was compared between the normal skin and rash, significantly higher concentrations of erlotinib were observed in the rash compared to normal skin tissue (1946 ± 1258 ng/cm^3^ in the rash vs. 1174 ± 662 ng/cm^3^ in the normal skin; *P* = 0.028, Figure 3) in the superficial skin layer (L1 with L2). In the deep skin layer (L3), there were no differences in erlotinib concentration between the rash and normal skin (1133 ± 518 and 1011 ± 697 ng/cm^3^ in the rash and normal skin, respectively; *P* = 0.214). LC-MS/MS and MALDI-MSI analyses were well correlated (coefficient of correlation 0.879, *P* < 0.0001) (Supplementary Figure 7).

## DISCUSSION

In this study, the difference in tissue concentration of erlotinib between skin rash and normal skin was documented in patients using both MALDI-MSI analysis as well as the combination of LMD and LC-MS/MS analysis. The results showed that the rash had a higher concentration of erlotinib than the adjacent normal skin in the certain sub-layer. Focal distribution analyses showed heterogeneous distribution of erlotinib within skin tissues; specifically, erlotinib was predominantly localized in the superficial layer of the skin tissue. Moreover, differences in erlotinib concentration between the normal skin and skin rash were observed in the superficial layer rather than in the deep layer.

Although the skin rash is the most common and cumbersome adverse effect of EGFR-TKI, it remains unknown if drug delivery and accumulation in the skin directly contributes to inflammation [6, 8, 13]. In this study, both MALDI-MSI and LC-MS/MS analyses showed that erlotinib was surely distributed in the patients’ skin tissues, and that the concentration of erlotinib was higher in the rash than in the adjacent normal skin. At least, the blood erlotinib concentration didn't seem to be higher than the paired tissue concentration (Supplementary Table 1). Although the molecular biological mechanism of underlying rash formation was not investigated in this clinical study, current data may suggest that daily erlotinib penetration and/or accumulation in focal skin tissue results in rash development. The pathogenesis of EGFR inhibitor-induced rash is not well clarified: however, certain inflammatory cytokines released by EGFR expressed keratinocytes can be involved with the rash formation [13]. The excess drug exposure to focal skin tissue may be an important factor for rash formation in the patients receiving EGFR-TKI. Future comparative studies between patients who developed skin rash and those maintain normal skin would help to clarify the effect of focal tissue concentrations of erlotinib on rash formation.

This study also showed that the epidermis and superficial dermis had higher concentrations of erlotinib than the deep dermis. It is possible that erlotinib distribution is dependent on the skin structure and blood circulation because the epidermis is the most outer layer and the superficial skin generally has rich micro-vessels. Whether erlotinib accumulation in the superficial skin involves binding to its target EGFR, which is predominantly expressed in the epidermis requires further study (Supplementary Figure 3). The evolution of measurement systems including MALDI-MSI [22] has enabled researchers to gain biological insights into the mechanism of action and toxicity of molecular-targeted compounds and biomarkers.

In clinical practice, the management of adverse effects is important to maintain the dose intensity of drug therapy for effective treatment and to increase the quality of life of patients. For example, a cooling cap reduces the frequency and severity of alopecia during cytotoxic chemotherapy [23] and frozen gloves reduce docetaxel-induced skin and nail toxicity [24]. These effects are achieved by reducing the concentrations of anti-cancer drugs in target tissues. Although the number of patients evaluated in this study was small, further study might support the development of methods to reduce the distribution of erlotinib into superficial skin for the prevention of skin rashes.

In conclusion, this study showed that the predominant distribution of erlotinib is in the superficial layer of skin rashes. Further studies on other EGFR-TKIs are required to determine if the same results are obtained, which would suggest that this phenomenon could be targeted to prevent adverse skin effects, thereby maximizing the benefits of therapy. The results from MALDI-MSI and conventional LC-MS/MS were well correlated, and may be useful for elucidating the mechanisms of action of molecular-targeted compounds and predicting their efficacy. The study with a further level of analysis with metabolomics or proteomics could enhance our results to examine molecular mechanisms. Advancements in imaging pharmacokinetic analyses using MALDI-MSI could accelerate the development of anticancer drugs and translational research.

## MATERIALS AND METHODS

### Study design, patient eligibility, and samples

Patients (≥20 years old) with histological or cytological evidence of unresectable locally advanced or metastatic pancreatic cancer (excluding neuroendocrine tumor) at Kobe University Hospital, Japan, were enrolled in this prospective study (UMIN000016297). Other major eligibility criteria were an Eastern Cooperative Oncology Group performance status of 0 or 1, and adequate hematologic, renal, and hepatic functions. Patients with dermatological disease such as atopic dermatitis or collagen disease were excluded. Patients were treated with erlotinib (given orally at 100 mg/day) and gemcitabine (1000 mg/m^2^ by intravenous infusion for 30 min on days 1, 8, and 15 every 4 weeks). The severity of skin rash was graded using the Common Terminology Criteria for Adverse Events. In patients who developed rash after erlotinib administration, we biopsied rash tissues concurrently with adjacent normal skin tissues from the upper back using the Biopsy Punch (BP-20F, φ 2.0 mm, Kai industries, Gifu, Japan) under local anesthesia (Supplementary Figure 1). At the same time of skin biopsy, we also collected a blood sample from each patient to measure erlotinib concentration in the plasma. The Institutional Review Boards of Kobe University Hospital and the National Cancer Center, Japan approved this study. All patients provided written informed consent, and the study was conducted in accordance with the principles of the Declaration of Helsinki. The methods of immunohistochemistry and histological evaluation are described in the Supplementary Data.

### MS analyses

The concentrations of erlotinib in tissue homogenates and plasma were measured using QTRAP 4500 (AB Sciex, Framingham, MA, USA) and QTRAP 5500 (AB Sciex), respectively (Supplementary Figures 8 and 9). The evaluation of erlotinib distribution in skin tissues was performed with the iMScope MALDI-MSI instrument (Shimadzu; Kyoto, Japan) with α-cyano-4-hydroxycinnamic acid used as the matrix reagent, as previously described (Supplementary Figure 10) [20]. Erlotinib-d6 was used as the exogenous internal standard, and erlotinib ion intensity was adjusted by the internal standard to determine the background ionization efficiency of the tissue. Scale bar of erlotinib molecular image was converted from absolute ion intensity to erlotinib concentration using the sum of ion intensity and the total quantity of erlotinib by LC-MS/MS analysis of the serial sections. Detailed methods are described in the Supplementary Data.

### Statistical analysis

The significance of differences between the matched pair samples was evaluated using the paired *t*-test, unless stated otherwise. *P* values less than 0.05 were considered statistically significant. JMP software version 12.0.1 for Mac (SAS Institute Japan, Tokyo, Japan) was used for these analyses.

## SUPPLEMENTARY MATERIALS FIGURES



## Abbreviations

EGFR-TKI: epidermal growth factor receptortyrosine kinase inhibitor
LC-MS/MS: liquid chromatography-tandem mass spectrometry
MSI: mass spectrometry imaging
MALDI: matrix-assisted laser desorption/ionization
LMD: laser microdissection

## References

[1] DiMasi JA, Reichert JM, Feldman L, Malins A (2013). Clinical approval success rates for investigational cancer drugs. Clin Pharmacol Ther.

[2] Waring MJ, Arrowsmith J, Leach AR, Leeson PD, Mandrell S, Owen RM, Pairaudeau G, Pennie WD, Pickett SD, Wang J, Wallace O, Weir A (2015). An analysis of the attrition of drug candidates from four major pharmaceutical companies. Nat Rev Drug Discov.

[3] Fujiwara Y (2016). Evolution of frameworks for expediting access to new drugs in Japan. Nat Rev Drug Discov.

[4] Harrison RK (2016). Phase II and phase III failures: 2013-2015. Nat Rev Drug Discov.

[5] Smietana K, Siatkowski M, Moller M (2016). Trends in clinical success rates. Nat Rev Drug Discov.

[6] Moore MJ, Goldstein D, Hamm J, Figer A, Hecht JR, Gallinger S, Au HJ, Murawa P, Walde D, Wolff RA, Campos D, Lim R, Ding K (2007). Erlotinib plus gemcitabine compared with gemcitabine alone in patients with advanced pancreatic cancer: a phase III trial of the National Cancer Institute of Canada Clinical Trials Group. J Clin Oncol.

[7] Shepherd FA, Rodrigues Pereira J, Ciuleanu T, Tan EH, Hirsh V, Thongprasert S, Campos D, Maoleekoonpiroj S, Smylie M, Martins R, van Kooten M, Dediu M, Findlay B (2005). Erlotinib in previously treated non-small-cell lung cancer. N Engl J Med.

[8] Okusaka T, Furuse J, Funakoshi A, Ioka T, Yamao K, Ohkawa S, Boku N, Komatsu Y, Nakamori S, Iguchi H, Ito T, Nakagawa K, Nakachi K (2011). Phase II study of erlotinib plus gemcitabine in Japanese patients with unresectable pancreatic cancer. Cancer Sci.

[9] Kubota K, Nishiwaki Y, Tamura T, Nakagawa K, Matsui K, Watanabe K, Hida T, Kawahara M, Katakami N, Takeda K, Yokoyama A, Noda K, Fukuoka M (2008). Efficacy and safety of erlotinib monotherapy for Japanese patients with advanced non-small cell lung cancer: a phase II study. J Thorac Oncol.

[10] Zhou C, Wu YL, Chen G, Feng J, Liu XQ, Wang C, Zhang S, Wang J, Zhou S, Ren S, Lu S, Zhang L, Hu C (2011). Erlotinib versus chemotherapy as first-line treatment for patients with advanced EGFR mutation-positive non-small-cell lung cancer (OPTIMAL, CTONG-0802): a multicentre, open-label, randomised, phase 3 study. Lancet Oncol.

[11] Wacker B, Nagrani T, Weinberg J, Witt K, Clark G, Cagnoni PJ (2007). Correlation between development of rash and efficacy in patients treated with the epidermal growth factor receptor tyrosine kinase inhibitor erlotinib in two large phase III studies. Clin Cancer Res.

[12] Lacouture ME (2006). Mechanisms of cutaneous toxicities to EGFR inhibitors. Nat Rev Cancer.

[13] Fabbrocini G, Panariello L, Caro G, Cacciapuoti S (2015). Acneiform Rash Induced by EGFR Inhibitors. Review of the Literature and New Insights. Skin Appendage Disord.

[14] Nilsson A, Goodwin RJ, Shariatgorji M, Vallianatou T, Webborn PJ, Andren PE (2015). Mass spectrometry imaging in drug development. Anal Chem.

[15] Wishart DS (2016). Emerging applications of metabolomics in drug discovery and precision medicine. Nat Rev Drug Discov.

[16] Castellino S, Groseclose MR, Wagner D (2011). MALDI imaging mass spectrometry: bridging biology and chemistry in drug development. Bioanalysis.

[17] Fuso Nerini I, Morosi L, Zucchetti M, Ballerini A, Giavazzi R, D’Incalci M (2014). Intratumor heterogeneity and its impact on drug distribution and sensitivity. Clin Pharmacol Ther.

[18] Cesca M, Morosi L, Berndt A, Fuso Nerini I, Frapolli R, Richter P, Decio A, Dirsch O, Micotti E, Giordano S, D’Incalci M, Davoli E, Zucchetti M (2016). Bevacizumab-induced inhibition of angiogenesis promotes a more homogeneous intratumoral distribution of paclitaxel, improving the antitumor response. Mol Cancer Ther.

[19] Connell JJ, Sugihara Y, Torok S, Dome B, Tovari J, Fehniger TE, Marko-Varga G, Vegvari A (2015). Localization of sunitinib in *in vivo* animal and *in vitro* experimental models by MALDI mass spectrometry imaging. Anal Bioanal Chem.

[20] Aikawa H, Hayashi M, Ryu S, Yamashita M, Ohtsuka N, Nishidate M, Fujiwara Y, Hamada A (2016). Visualizing spatial distribution of alectinib in murine brain using quantitative mass spectrometry imaging. Sci Rep.

[21] Ashton S, Song YH, Nolan J, Cadogan E, Murray J, Odedra R, Foster J, Hall PA, Low S, Taylor P, Ellston R, Polanska UM, Wilson J (2016). Aurora kinase inhibitor nanoparticles target tumors with favorable therapeutic index *in vivo*. Sci Transl Med.

[22] Kompauer M, Heiles S, Spengler B (2017). Atmospheric pressure MALDI mass spectrometry imaging of tissues and cells at 1.4-mum lateral resolution. Nat Methods.

[23] Rugo HS, Klein P, Melin SA, Hurvitz SA, Melisko ME, Moore A, Park G, Mitchel J, Bageman E, D’Agostino RB, Ver Hoeve ES, Esserman L, Cigler T (2017). Association Between Use of a Scalp Cooling Device and Alopecia After Chemotherapy for Breast Cancer. JAMA.

[24] Scotte F, Tourani JM, Banu E, Peyromaure M, Levy E, Marsan S, Magherini E, Fabre-Guillevin E, Andrieu JM, Oudard S (2005). Multicenter study of a frozen glove to prevent docetaxel-induced onycholysis and cutaneous toxicity of the hand. J Clin Oncol.

