# Supplementary material for: Distribution of erlotinib in rash and normal skin in cancer patients receiving erlotinib visualized by matrix assisted laser desorption/ionization mass spectrometry imaging
Source: Oncotarget. 2018 Apr 6;9(26):18540–7. doi: 10.18632/oncotarget.24928 (PMC5915091; doi:10.18632/oncotarget.24928)
Supplement: Supplementary file 1 [file oncotarget-09-18540-s001.pdf]

Distribution of erlotinib in rash and normal skin in cancer patients receiving erlotinib visualized by matrix assisted laser desorption/ ionization mass spectrometry imaging

SUPPLEMENTARY MATERIALS

Sample preparation

The tissue samples were flash-frozen in vapor-phase liquid N_2 without an embedding agent, and were then stored at –80° C until subsequent analysis. At the time of tissue biopsy, a blood samples was collected from each patient in heparin-containing tubes, and centrifuged at 1500 × *g* at 4° C for 10 min to obtain the plasma, which was stored at –80° C until subsequent analysis. The frozen tissues were sliced into 8 μm thick slices at −20° C with a cryomicrotome (Leica CM 1950, Tokyo, Japan) into at least six serial sections, with the exception of one patient's paired samples (rash and normal skin tissues) that was insufficient in quantity for laser microdissection (LMD) and was cut into three serial sections. The first and third sections were used for liquid chromatography-tandem mass spectrometry (LC-MS/MS) measurement of erlotinib concentration in the tissue, whereas the second section was placed on indium tin oxide-coated (ITO) glass slides (Matsunami Glass Ind., Ltd., Tokyo, Japan) and used for matrix-assisted laser desorption/ionization mass spectrometry imaging (MALDI-MSI). The BZ-X710 microscope (Keyence, Itasca, IL, USA) was used for the observation and calculation of the target tissue and infiltrating inflammatory cells areas. The fourth to sixth tissue sections were used for LC-MS/MS of erlotinib concentration in the small pieces that were cut off by LMD (Leica LMD 6500).

LC-MS/MS

Erlotinib was extracted from the plasma by solid phase extraction using Oasis HLB 96-well plates (10 mg sorbent per well, Waters, Milford, MA, USA). Erlotinib calibration standards ranging from 5 to 5000 ng/mL (linearity, R^2 = 0.9988) were prepared from standard solutions with human pooled plasma (Sigma-Aldrich, St. Louis, MO, USA), and used during the measurement of plasma erlotinib concentration (Supplementary Figure 8, available online). Erlotinib-d6 was used as the internal standard (100 ng/mL). For erlotinib extraction from tissues, the sections were vortexed for 30 s in 100 μL of 30% acetonitrile, 10% isopropanol, and 0.1% formic acid (FA), and centrifuged at 120,000 × *g* for 10 min at 4° C, after which the supernatants were collected. A total of 20 μL supernatants and internal standard, 260 μL of 50% methanol and 0.1% FA were used for the quantitation of erlotinib, with calibration standards ranging from 0.5 to 100 ng/mL (linearity R^2 = 0.9994) (Supplementary Figure 9, available online). Chromatographic separation of the erlotinib and erlotinib-d6 was performed using the XBridge C18 HPLC column (2.1 × 5.0 mm, 3.5 mm, Waters) maintained at 40° C. Mobile phases A and B consisted of 0.1% FA aqueous solution and acetonitrile containing 0.1% FA, respectively. Separation was performed using a gradient elution at a flow rate of 0.2 mL/min with a Nexera X2 series (Shimadzu, Co., Kyoto, Japan). The gradient elution of the two mobile phase systems with 0.1% FA aqueous (A) and acetonitrile containing 0.1% FA (B) was as follows: 20–65% B for 0–4.0 min, 65–90% B for 4.0–4.1 min, 90% B for 4.1–5.1 min and 90–20% B for 5.1–5.2 min, with a re-equilibrium time of 3 min. The column was equilibrated with the mobile phase for 10 min, with a run time of 8 min for each 1 μL (plasma sample) or 20 μL (tissue sample) injection volume. Quantitation was conducted by selected reaction monitoring on a QTRAP5500 (plasma sample) or QTRAP4500 (tissue sample) mass spectrometer (AB Sciex, Framingham, MA, USA) with electrospray ionization in the positive mode. The optimized electrospray ionization parameters were as follows: ion source temperature, 600° C; curtain gas, setting 50; nebulizing gas (GS1), setting 70; turbo-ionspray gas (GS2), setting 80; ion spray voltage, 5000 V; declustering potential, 6 V; and collision energy, 33V. The selected reaction monitoring transitions were m/z 394.0 to 336.0 for erlotinib and m/z 400.0 to 338.9 for erlotinib-d6. The dwell time was 500 ms for each transition channel. All data were acquired and analyzed using the Analyst version 1.6.1 software (AB Sciex).

MALDI-MSI

α-cyano-4-hydroxycinnamic acid (CHCA, Sigma) was coated on the dried section placed on the ITO glass slide using a vacuum sublimation apparatus (SVC-

700TMSG/7PS80, SANYU Electron, Tokyo, Japan) at 250° C for 8 min, after which 10 mg/mL CHCA solution containing 30% acetonitrile, 10% isopropanol, and 0.1% FA was sequentially sprayed on the section using a sprayer (PS270, GSI Creos, Tokyo, Japan) in the following 10 cycles; the first three cycles were for 3 s with spraying at 90 s intervals, and the next seven cycles were for 1 s with spraying at 30 s intervals. To evaluate the ionization efficiency of erlotinib on the tissue sections and for adjustment of ionization efficiency between samples, erlotinib-d6 was used as the internal standard in the CHCA solution (2 mg/mL). MALDI-MSI was performed using the iMScope (Shimadzu; Kyoto, Japan), which consisted of an optical microscope and a quadrupole ion trap time-of-flight analyzer with an atmospheric pressure MALDI source consisting of a 335 nm Nd:YAG laser (minimum laser spot diameter <10 μm). The spatial resolution of this examination was at a step size of 60 mm (Supplementary Figure 10, available online), and the erlotinib-d6 image was acquired with a 30 mm shift from the laser spot obtained with erlotinib. The optimized laser parameters were as follows: strength, setting 40; spot size, setting 2; number of shot, 50 shots; and frequency, 1,000 Hz. The optimized detector parameters were as follows; ion mode, positive; accumulation, 3 times/ pixel; sample voltage, 3.5 kV; detector voltage, 2.1 kV; MS/MS energy, 50%; gas volume, 50%, isolation time, 20 ms; and collision-induced dissociation time, 30 ms. Molecular images of erlotinib and erlotinib-d6 were acquired in tandem mass spectrometry and the optimal transitions were m/z 394.10 ± 3.0 $[M + H]^+$ to 336.15 ± 0.05 (mass range, m/z 100 to 396) for erlotinib, and m/z 400.10 ± 1.5 $[M + H]^+$ to 339.15 ± 0.05 (mass range, m/z 100 to 405) for erlotinib-d6. Raw molecular images of erlotinib and erlotinib-d6 were visualized using Imaging MS Solution (version 1.11, Shimadzu) with an absolute ion intensity scale (Supplementary Figure 4, available online). Biomap version 3.8.0.4 (Novartis Institutes for BioMedical Research, Basel, Switzerland) was used to display the adjusted molecular image of erlotinib distribution (divided images of raw erlotinib image by erlotinib-d6 image). Scale bar of erlotinib molecular image was converted from absolute ion intensity to erlotinib concentration using the sum of ion intensity and the total quantity of erlotinib by LC-MS/MS analysis of the serial sections. We also employed Biomap software to extract the ion data in the regions of interests. Mass spectrometric experiments were partly performed by Shimadzu Techno-Research (Tokyo, Japan).

Immunohistochemistry and histological evaluation

Hematoxylin and eosin staining of tissue section was used for the observation of histological finding and calculation of the infiltrating inflammatory cells areas. Epidermal growth factor receptor (EGFR) staining was performed with BenchMark XT (Ventana Medical Systems/Roche Diagnostics, Tucson AZ, USA) using *ultra*VIEW Universal DAB Detection Kit (Ventana/Roche) according to the manufacturer's instructions with EGFR (3C6) antibody (Ventana/Roche) (Supplementary Figure 3, avaiable online).

Supplementary Table 1: Characteristics of patients and erlotinib concentrations by liquid chromatography-tandem mass spectrometry

Patient list	Age (yrs)	Rash grade[a]	Duration time[b] (month)	Time after administration[c] (hour)	Treatment efficacy	Erlotinib concentrations			Sample volume
						Plasma (ng/mL)	Entire section (ng/cm^3)		
							Normal	Rash	
1	55	2	3	6	SD[d]	647	3441	5134	Low
2	51	1	4	6	SD	843	1343	1523	Sufficient
3	65	1	1	2	PD[e]	2640	2333	2775	Sufficient
4	53	2	2	3	SD	1270	1073	2090	Sufficient
5	70	2	0.75	3	PD	2580	4551	4377	Sufficient

[a]According to Common Terminology Criteria for Adverse Events (CTCAE), version 4.0 [b]Duration of the treatment to biopsy [c]Elapsed time to biopsy after latest oral administration of erlotinib [d]Stable Disease, [e]Progressive Disease by Response Evaluation Criteria in Solid Tumors (RECIST), version 1.1.

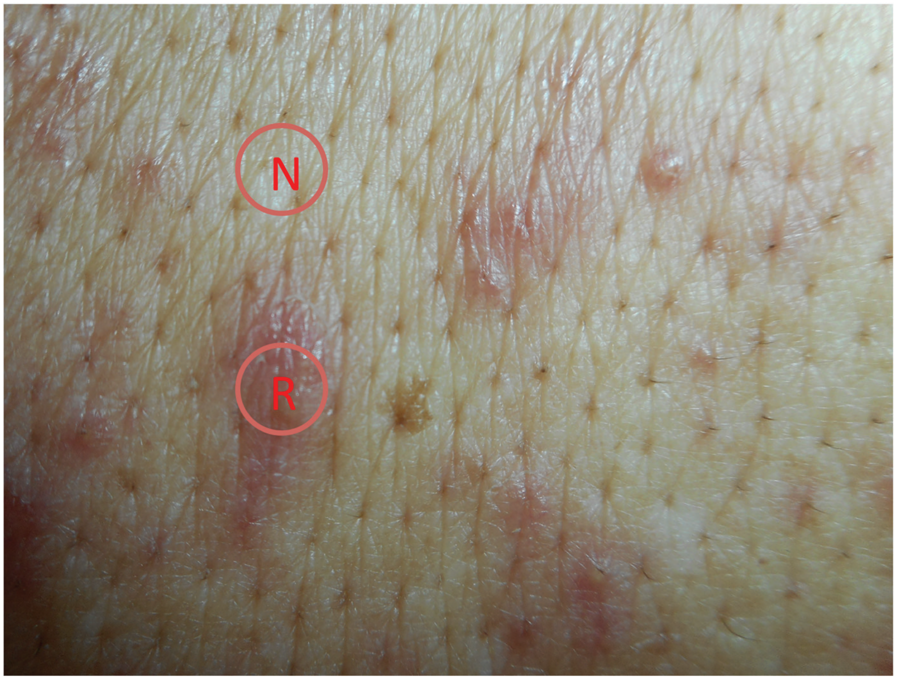

Supplementary Figure 1: Representative picture of erlotinib-associated rashes. Patients who developed rash after consecutive administration of erlotinib had their rashes (R), and adjacent normal skin (N) biopsied using Biopsy Punch (φ 2.0 mm).

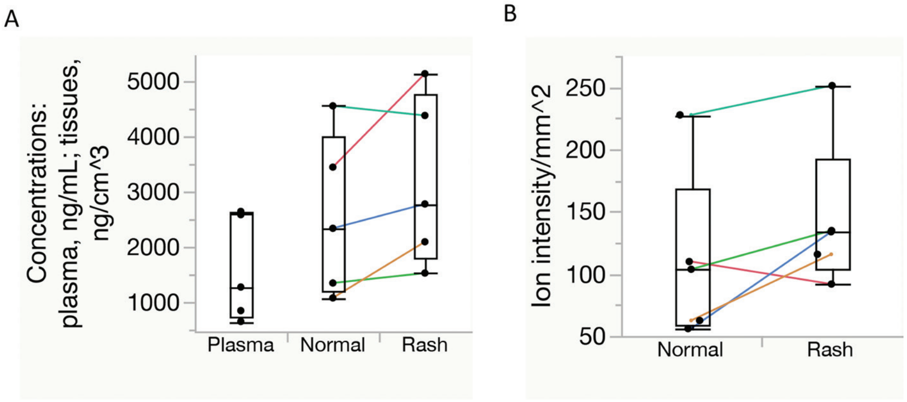

Supplementary Figure 2: Comparisons of erlotinib concentrations in entire tissue sections. (**A**) Erlotinib concentrations in plasma and skin tissues were measured by liquid chromatography-tandem mass spectrometry. Plasma and tissue samples were concurrently collected from each patient, who had a variety of time schedules of erlotinib administration. Each line indicates each patient. Units: plasma, ng/mL; tissues, ng/cm^3. (**B**) Erlotinib concentration in the entire tissue section was measured using mass spectrometry imaging and was compared between the normal skin and rash.

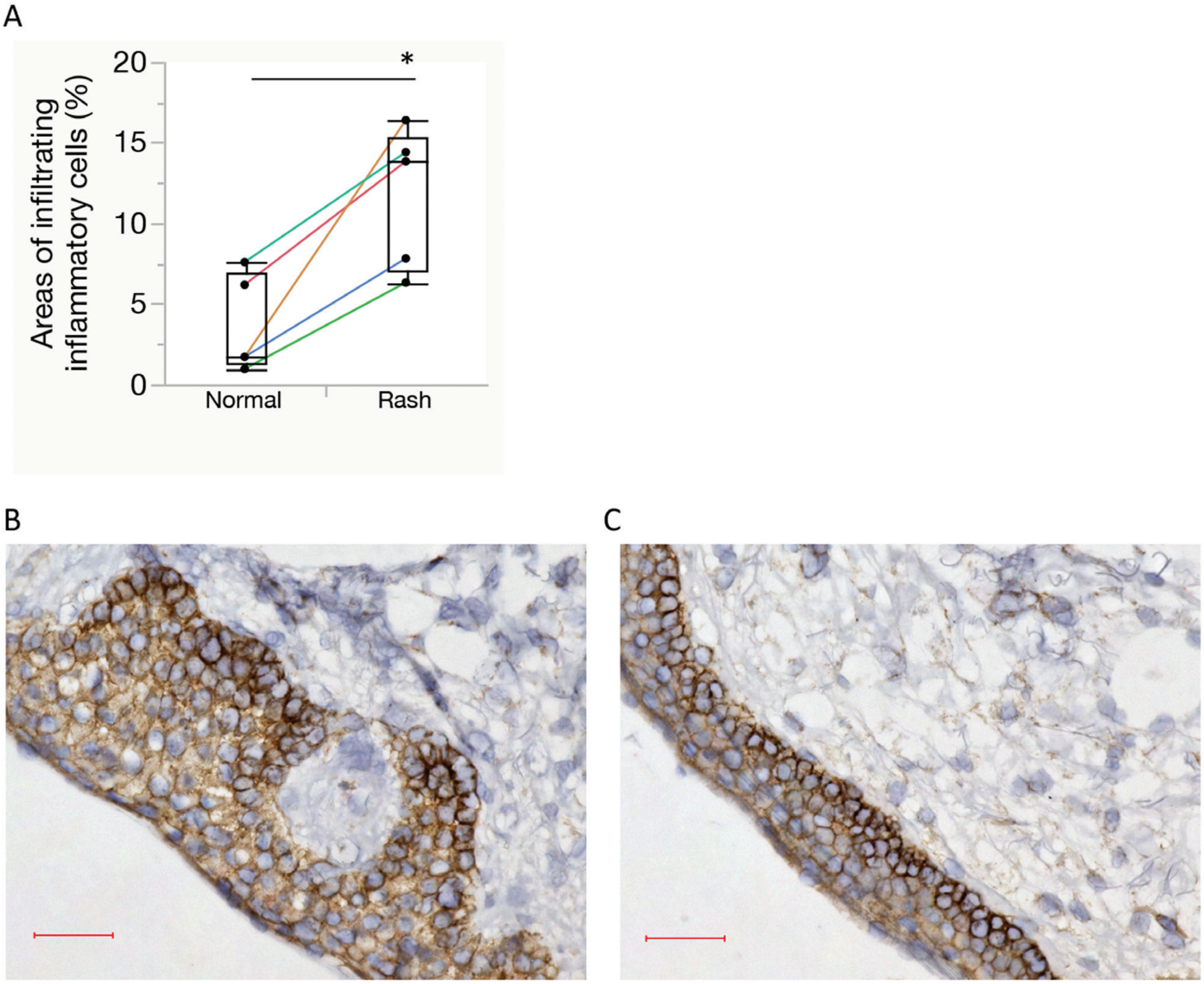

Supplementary Figure 3: Histological findings in the skin tissues. (**A**) The proportion of area of infiltrating inflammatory cells in the dermis was significantly larger in the rash than in the adjacent normal skin. Each line indicates each patient. $^{*}P < 0.005$. (**B**, **C**) Immunohistochemistry images of epidermal growth factor receptor (EGFR) of a rash and normal skin from the same patient. The rash showed thickened epidermis and irregular elongation of rete ridge. EGFR was predominantly expressed in the epidermis layer, and there was no obvious difference in EGFR expression between the rash and normal skin. Scale bar = 50 μm.

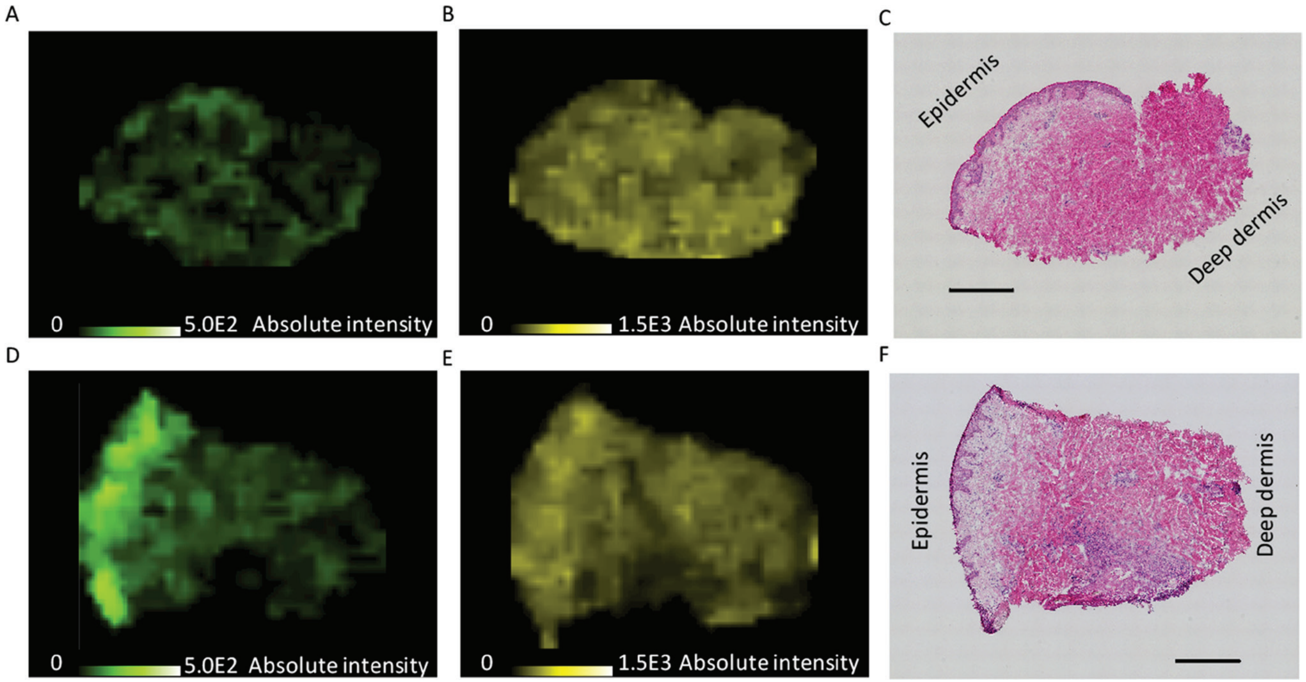

Supplementary Figure 4: Comparison of erlotinib distribution between adjacent normal skin and skin rash. (**A**) Raw molecular image of erlotinib distribution in adjacent normal skin using matrix-assisted laser desorption ionization mass spectrometry imaging. Scale bar indicates absolute ion intensities of erlotinib. (**B**) Molecular image of the exogenous internal standard, erlotinib-d6, in the normal skin shows background of erlotinib ionization efficiency of the tissue. (**C**) Hematoxylin and eosin staining of the normal skin using serial section. Scale bar = 500 µm. (**D**) Raw molecular image of erlotinib in a rash of same patient. Scale bar indicates absolute ion intensities of erlotinib. (**E**) Molecular image of the exogenous internal standard, erlotinib-d6, in a rash. (**F**) Hematoxylin and eosin staining of the rash using serial section. Scale bar = 500 µm. Molecular images were acquired at a step size of 60 µm, which was generated with the optimal transitions of erlotinib and erlotinib-d6, m/z 394.10 ($[M+H]^+$) ± 3.00 to 336.15 ± 0.05, and m/z 400.10 ($[M+H]^+$) ± 1.50 to 339.15 ± 0.05, respectively. Images were visualized using Imaging MS Solution (version 1.11, Shimadzu) with image correction by median filter.

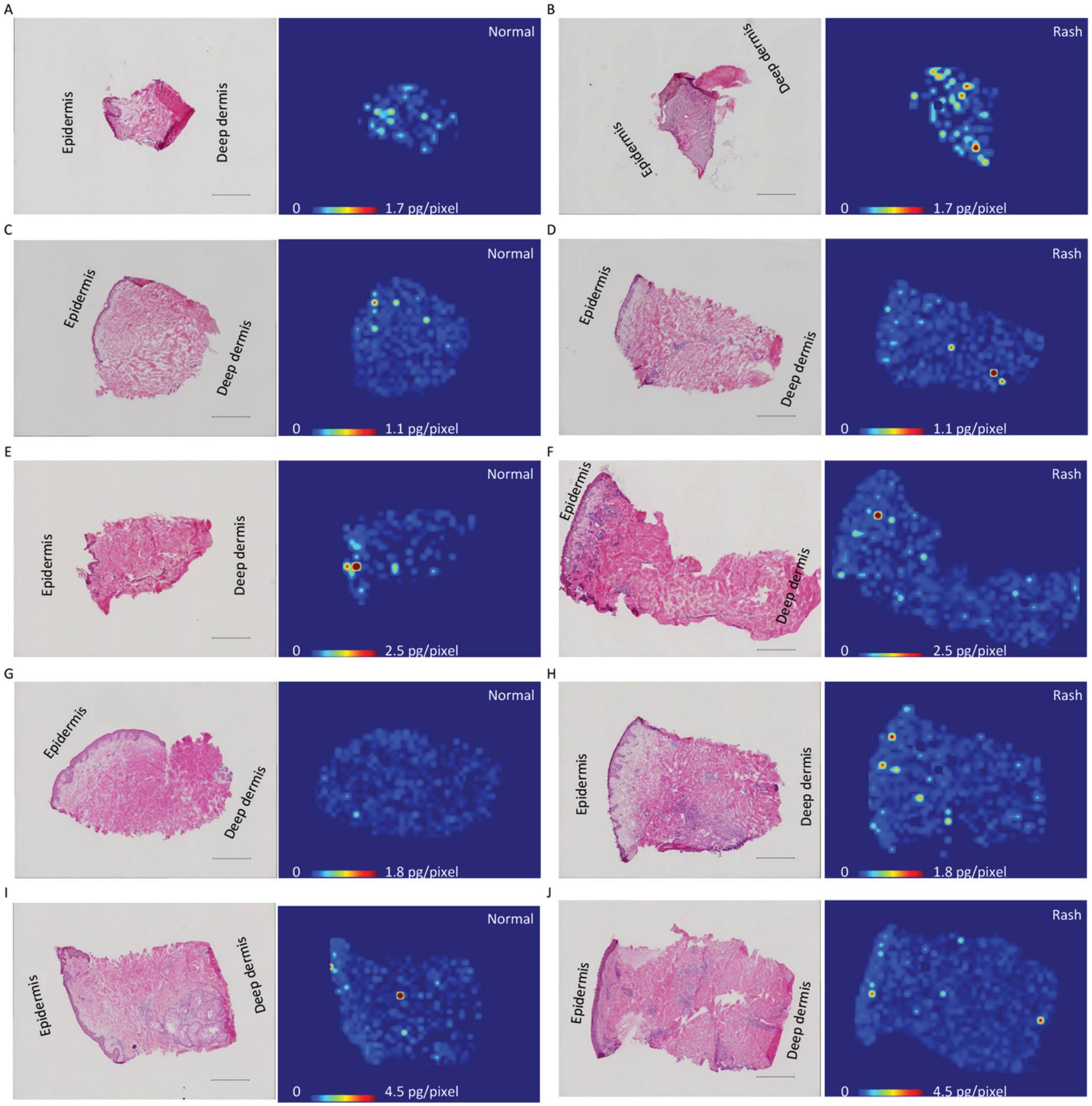

Supplementary Figure 5: Molecular images of erlotinib distribution in skin rash and adjacent normal skin of patients in this study. (**A**, **B**) The adjacent normal skin, A, and the rash, B, of patient #1. Left panel is hematoxylin and eosin staining, and right panel is molecular image of erlotinib distribution of each figure. These pair samples were excluded from focal distribution analysis because of insufficient tissue volume. (**C**, **D**) The adjacent normal skin, C, and the rash, D, of patient #2. Left panel is hematoxylin and eosin staining, and right panel is molecular image of erlotinib distribution of each figure. (**E**, **F**) The adjacent normal skin, E, and the rash, F, of patient #3. Left panel is hematoxylin and eosin staining, and right panel is molecular image of erlotinib distribution of each figure. (**G**, **H**) The adjacent normal skin, G, and the rash, H, of patient #4. Left panel is hematoxylin and eosin staining, and right panel is molecular image of erlotinib distribution of each figure. (**I**, **J**) The adjacent normal skin, I, and the rash, J, of patient #5. Left panel is hematoxylin and eosin staining, and right panel is molecular image of erlotinib distribution of each figure. Scale bar of hematoxylin and eosin staining = 500 µm. Scale bar of molecular image indicates erlotinib quantity, pg/pixel and was aligned between pairs of each patient.

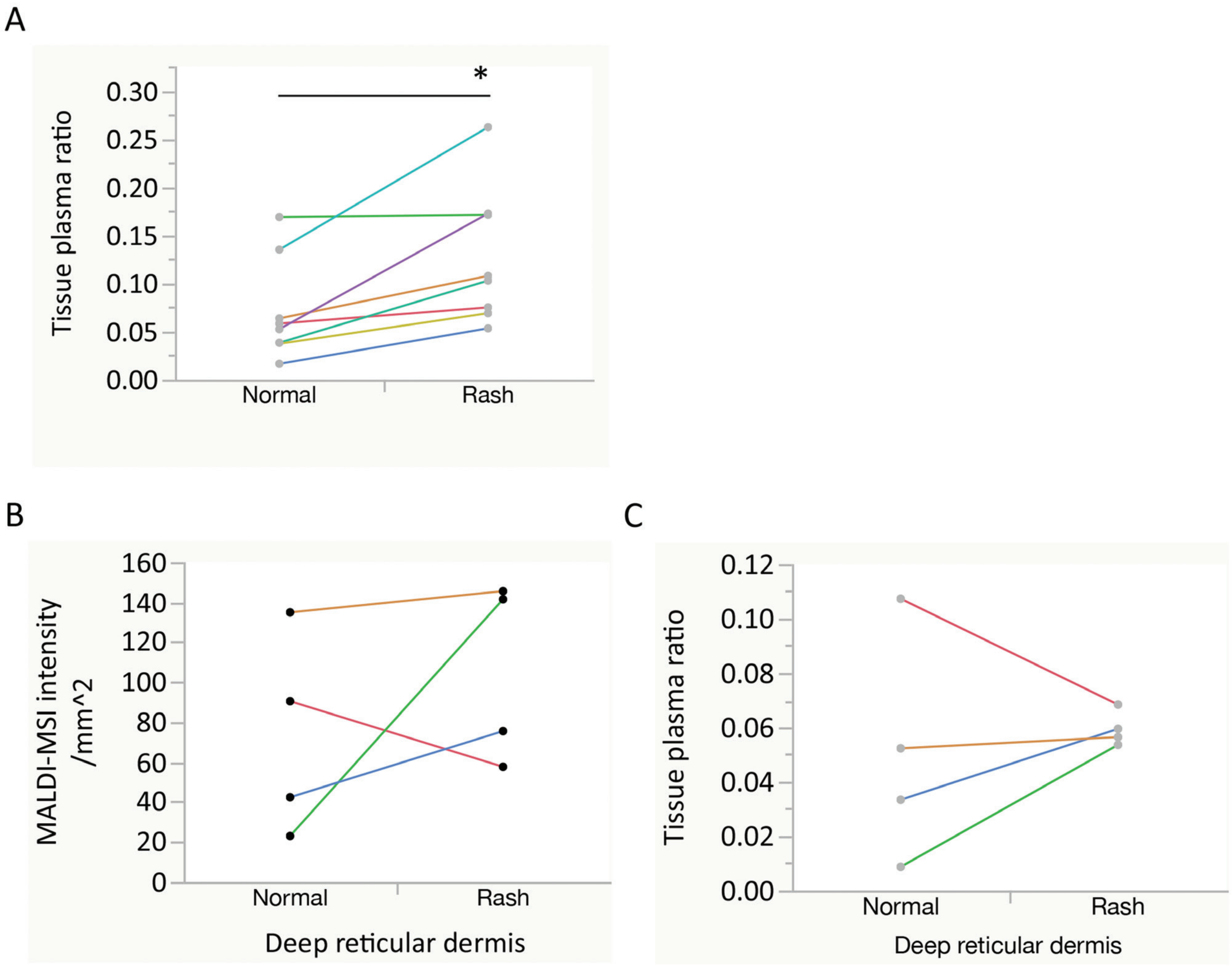

Supplementary Figure 6: Comparison of erlotinib focal concentration using mass spectrometry imaging. (**A**) Tissue plasma ratio, which is the relative value of erlotinib concentration of the tissue divided by the matched plasma concentration, was compared between the normal skin and rash in the superficial skin layer (epidermis–papillary dermis with superficial reticular dermis). Each line indicates each sample. $^{*}P < 0.05$. (**B**) Erlotinib concentrations were compared between the normal skin and rash in the deep skin layer (deep reticular dermis). (**C**) Tissue plasma ratio in deep skin layer was compared between the normal skin and rash.

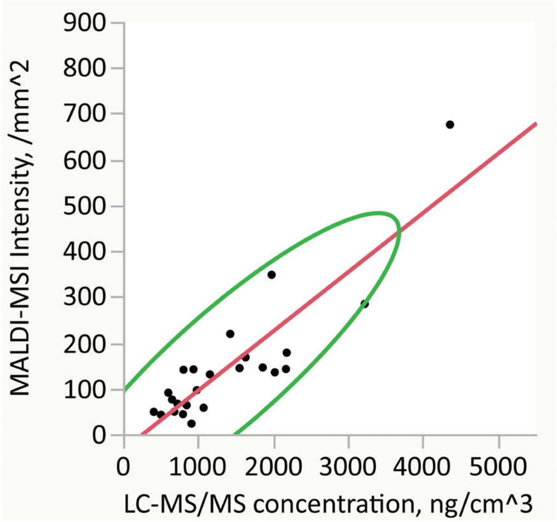

Supplementary Figure 7: Correlation between erlotinib concentrations measured with mass spectrometry imaging and liquid chromatography-tandem mass spectrometry. Erlotinib focal concentration measured with mass spectrometry imaging and erlotinib concentrations in the corresponding laser microdissected pieces measured by liquid chromatography-tandem mass spectrometry were well correlated (coefficient of correlation 0.879, $P < 0.0001$).

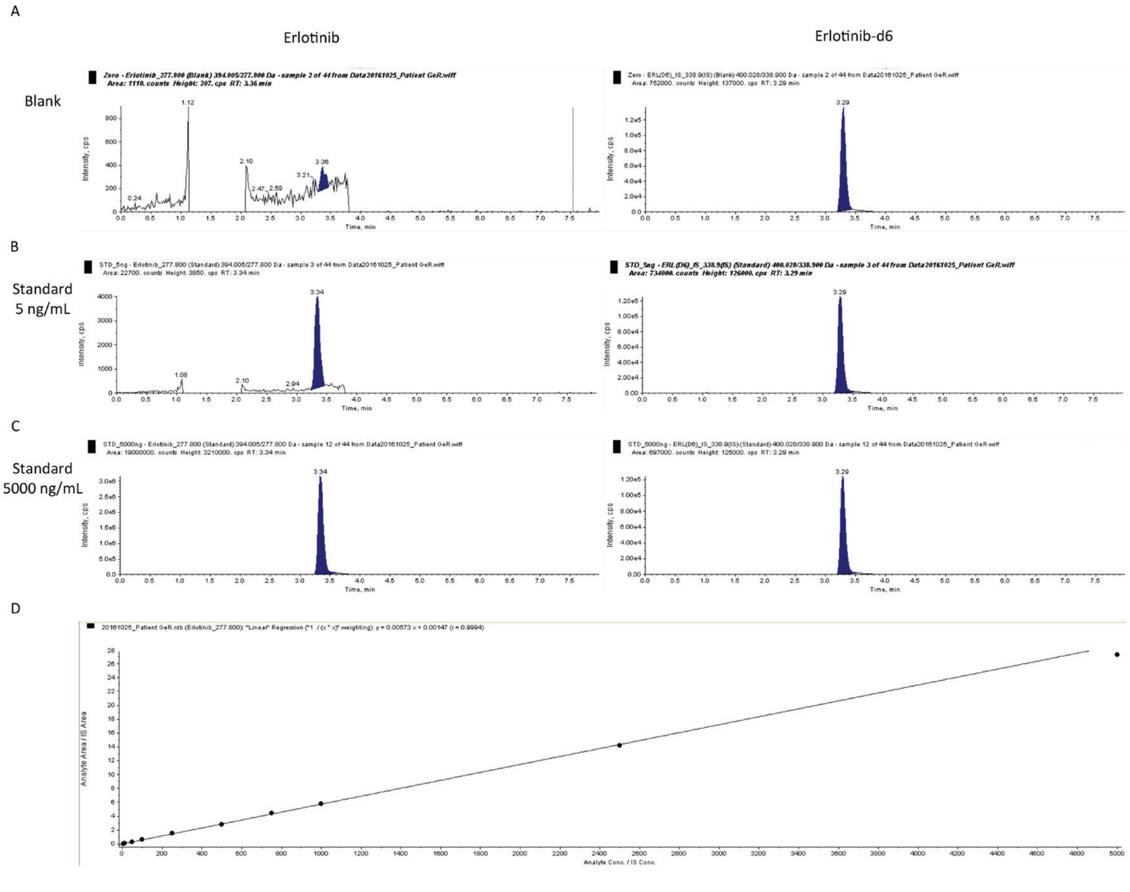

Supplementary Figure 8: Standard curve of erlotinib for liquid chromatography-tandem mass spectrometry in plasma sample. Erlotinib and erlotinib-d6 spectra of (**A**) blank sample, (**B**) standard sample of 5 ng/ml, and (**C**) 5000 ng/mL, respectively. (**D**) Standard curve ranging from 5 to 5000 ng/mL, $R^2 = 0.9988$.

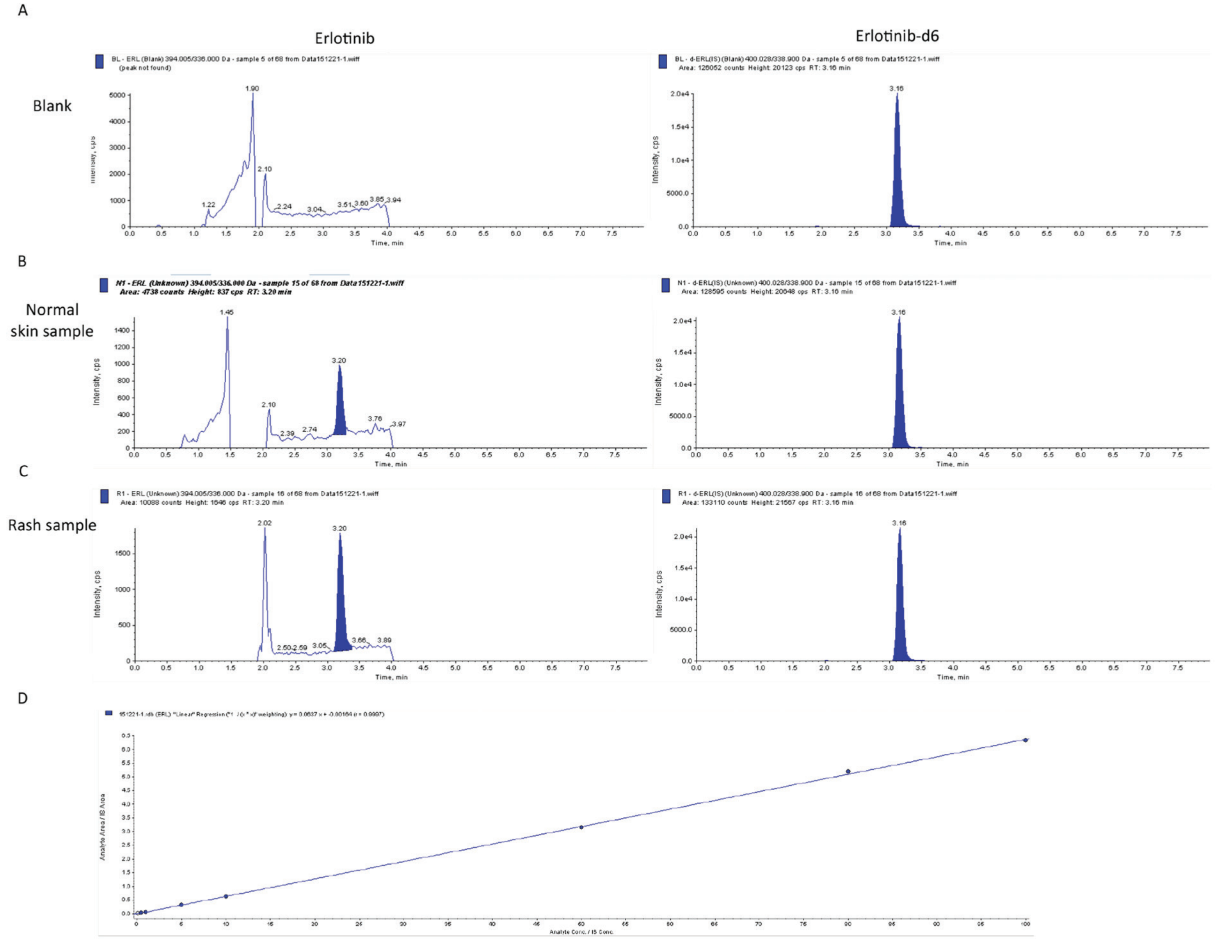

Supplementary Figure 9: Representative spectra in normal skin and a rash in liquid chromatography-tandem mass spectrometry. Erlotinib and erlotinib-d6 spectra of (**A**) blank sample, (**B**) normal skin, and (**C**) a rash, respectively. (**D**) Standard curve ranging from 0.5 to 100 ng/mL, $R^2 = 0.9994$.

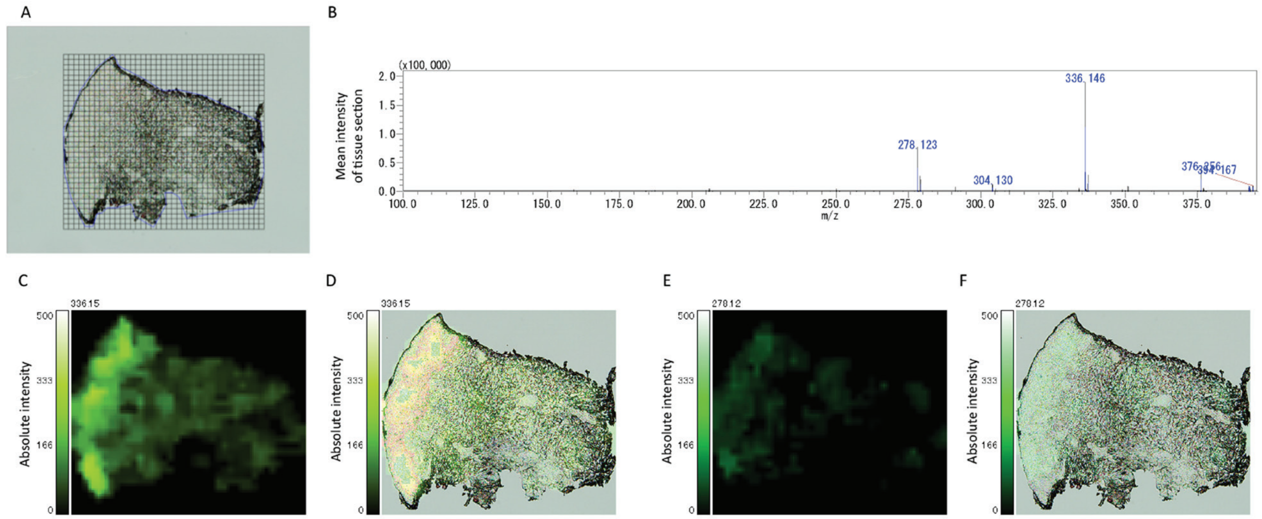

Supplementary Figure 10: MS/MS spectra and molecular images of erlotinib in matrix-assisted laser desorption/ ionization mass spectrometry imaging. (**A**) Erlotinib tissue distribution was examined by the spatial resolution of 60 mm step size using iMScope that consisted of an optical microscope. (**B**) MS/MS spectrum of the representative tissue. (**C**, **D**) Product ion image of 394.10 $[M + H]^+$ to 336.15 ± 0.05, and fusion image with optical tissue figure, respectively. (**E**, **F**) Product ion image of 394.10 $[M + H]^+$ to 278.12 ± 0.05, and fusion image with optical tissue figure, respectively.
